# Neurophysiological Factors Affecting Muscle Innervation Zone Estimation Using Surface EMG: A Simulation Study

**DOI:** 10.3390/bios11100356

**Published:** 2021-09-27

**Authors:** Chengjun Huang, Maoqi Chen, Xiaoyan Li, Yingchun Zhang, Sheng Li, Ping Zhou

**Affiliations:** 1 Guangdong Work Injury Rehabilitation Center, Guangzhou 510970, China; ahhcj123@gmail.com; 2 Department of Physical Medicine and Rehabilitation, University of Texas Health Science Center at Houston, Houston, TX 77030, USA; Sheng.Li@uth.tmc.edu; 3 Faculty of Rehabilitation Engineering, University of Health and Rehabilitation Sciences, Qingdao 266024, China; maoqi.chen@uor.edu.cn; 4 Department of Bioengineering, University of Maryland, College Park, MD 20742, USA; xli025@umd.edu; 5 Department of Neurology, Medical College of Wisconsin, Milwaukee, WI 53226, USA; 6 Department of Biomedical Engineering, University of Houston, Houston, TX 77204, USA; yzhang94@uh.edu

**Keywords:** surface electromyography, innervation zone, motor unit synchronization, M wave, electrode array, simulation

## Abstract

Surface electromyography (EMG) recorded by a linear or 2-dimensional electrode array can be used to estimate the location of muscle innervation zones (IZ). There are various neurophysiological factors that may influence surface EMG and thus potentially compromise muscle IZ estimation. The objective of this study was to evaluate how surface-EMG-based IZ estimation might be affected by different factors, including varying degrees of motor unit (MU) synchronization in the case of single or double IZs. The study was performed by implementing a model simulating surface EMG activity. Three different MU synchronization conditions were simulated, namely no synchronization, medium level synchronization, and complete synchronization analog to M wave. Surface EMG signals recorded by a 2-dimensional electrode array were simulated from a muscle with single and double IZs, respectively. For each situation, the IZ was estimated from surface EMG and compared with the one used in the model for performance evaluation. For the muscle with only one IZ, the estimated IZ location from surface EMG was consistent with the one used in the model for all the three MU synchronization conditions. For the muscle with double IZs, at least one IZ was appropriately estimated from interference surface EMG when there was no MU synchronization. However, the estimated IZ was different from either of the two IZ locations used in the model for the other two MU synchronization conditions. For muscles with a single IZ, MU synchronization has little effect on IZ estimation from electrode array surface EMG. However, caution is required for multiple IZ muscles since MU synchronization might lead to false IZ estimation.

## 1. Introduction

The site where the terminal branches of a motor neuron contact the muscle fibers is referred to as neuromuscular junction. These junctions tend to cluster in a relatively narrow band, which is termed the innervation zone (IZ). Identification of muscle IZ is of great importance for both basic and clinical investigations. For example, how IZ is distributed is an essential component for understanding muscle morphology and its alterations in diseased states [1,2,3,4,5]. In clinical practice, identification of muscle IZ can help facilitate botulinum toxin injection for spasticity treatment [6,7,8,9]. Knowledge of muscle IZ is also helpful for episiotomy during child delivery to minimize the risk of sphincter denervation [10]. Estimation of muscle IZ is also useful in guiding appropriate electrode placement for surface electromyogram (EMG) recording [11,12,13,14,15,16,17], although an area has been suggested in previous literature favorable for appropriate positioning of an electrode pair without prior knowledge of IZ location [11].

When a motor neuron is excited, motor unit (MU) action potentials (MUAPs) originate from the neuromuscular junctions and propagate in opposite directions toward the muscle tendons. Surface EMG signals recorded by a linear electrode array or a matrix of electrodes can be used to estimate muscle IZ location [18,19,20]. For a bipolar or single differential electrode configuration along muscle fibers, the IZ can be likely estimated with visual inspection to be closest to the channel having the minimum surface EMG amplitude or between the two adjacent channels showing action potential phase reversals. When visual inspection is difficult, the IZ can be estimated by computer-based methods, for example, calculating correlation coefficients and searching the minimum one between every two adjacent surface EMG channels along muscle fibers [18]. In fact, various methods have been developed to improve muscle IZ detection from electrode array surface EMG, using signal processing techniques including Radon transform, optical flow, linear regression, three-dimensional imaging, etc. [21,22,23,24,25,26,27,28,29]. Most of studies use interference surface EMG from voluntary contractions to perform IZ estimation. An alternative method is to apply electrical stimulation to evoke compound muscle action potentials or M waves, which is often used for those who have difficulty performing voluntary contractions because of paralysis or poor motor control [30].

When estimating the IZ of a muscle using surface EMG, it usually assumes that the neuromuscular junctions cluster in a single narrow band, forming only one IZ. However, it is also possible that a muscle may have neuromuscular junctions clustered in different regions, forming multiple IZs [31]. For example, in an early study, Masuda et al. showed that the biceps brachii had two IZs [32]. More recently, Beretta-Piccoli et al. investigated 43 superficial muscles and identified multiple IZs in the biceps brachii and brachioradialis muscles of some participants (6–13 out of 40) [11]. In reference [1], the authors identified multiple IZs in pronator teres and rectus femoris muscles of few participants. These reports indicate that there is a possibility of a superficial muscle having multiple IZs, although the possibility is relatively low, or at least not related to all the individuals. For these muscles, surface EMG from different IZs can propagate in opposite directions, which may have a significant effect on the recorded surface EMG. Another factor that may influence surface EMG is MU synchronization [33], referred to as a significant coincidence in the relative timing of discharges between pairs of MUs due to the presence of common synaptic inputs to MUs, modulations in the muscle-afferent feedback, byproduct of other physiological mechanisms, or an epiphenomenon of the MU firing characteristics [34,35,36,37,38]. It has been reported that MU synchronization tends to increase the amplitude of the EMG and decrease the steadiness of the force exerted by the muscle [39].

Although the presence of multiple IZs and MU synchronization has a significant effect on surface EMG generation, it remains unclear how these neurophysiological factors will influence surface-EMG-based IZ identification. The purpose of this study was, therefore, to evaluate the performance of IZ estimation under different situations with respect to these neurophysiological factors. Given that these factors are difficult to adjust experimentally, a simulation approach was used in this study. Surface EMG signals recorded by a two-dimensional electrode array were simulated from muscles having one or two IZs. For each muscle, three different conditions of MU synchronization were simulated, namely no synchronization, medium level synchronization during voluntary contraction, and complete synchronization with all MUs firing simultaneously, analog to maximum M wave or compound muscle action potential evoked by supramaximal electrical stimulation. For each of the combined situations, the IZ was estimated from simulated electrode array surface EMG and compared with the one used in EMG simulation for performance evaluation. The findings of this study can help understand the effects of complex neurophysiological factors on surface-EMG-based IZ estimation in order to be aware and cautious about the pitfalls during IZ estimation using surface EMG.

## 2. Materials and Methods

The model used in this study contained two components: an MU pool activation model describing MU recruitment and firing rates, and a surface EMG model describing recording and generation of surface EMG signals. The setting of the model parameters primarily followed reference [40].

### 2.1. Motor Unit Pool Simulation

A total of 120 MUs were included in the MU pool [40]. The recruitment threshold (*RTE*) of each MU was expressed as an exponential function as Equation (1), where *RR* represents the *RTE* range between the first and last MUs in the pool, and *i* is an index identifying each MU. *RR* was assigned to be 40% excitation (i.e., the last MU was recruited at 40% maximum excitation).

(1) $$RTE\left(i\right)=e^{\left(\frac{lnRR}{n}\right)\;i}$$

The minimum firing rate (MFR) of all the MUs was set at 8 Hz when the excitatory drive reached the *RTE*. MU firing rate (FR) increased linearly with the excitatory drive until the peak firing rate (PFR) was reached, as expressed in Equation (2), where G is the gain between the FR and excitatory input, which was the same for all MUs (gain = 30). In this study, all MUs followed the “onion skin” firing strategy, i.e., the PFR of each MU was inversely proportional to its *RTE*. The PFRs of later recruited large MUs were lower than the early recruited small ones. Therefore, the PFR of the largest MU in the study was 25, and the PFR of the smallest MU was 35. To simulate the stochastic nature of motor neuron discharge, the inter-spike interval of the MU firing was modeled as a random process with a Gaussian probability distribution function. The standard deviation of the inter-spike interval was fixed for all MUs at 20% of the mean inter-spike interval.

(2) FRi= G·[E(t)−RTEi]+ MFR   E(t) ≥RTE

### 2.2. Surface EMG Simulation

The shape of the muscle was cylindrical, and the radius was 8 mm. The thickness of fat and skin layers was 2.5 mm. There were totally 70,000 muscle fibers innervated by 120 MUs. Following an exponential function, the number of muscle fibers of each MU was simulated to have a wide range as 100-fold [40]. The fibers of each MU were randomly scattered in a circular territory and distributed in parallel. The density of the circular territory was approximately 20 fibers/mm^2^.

**Figure 1:**
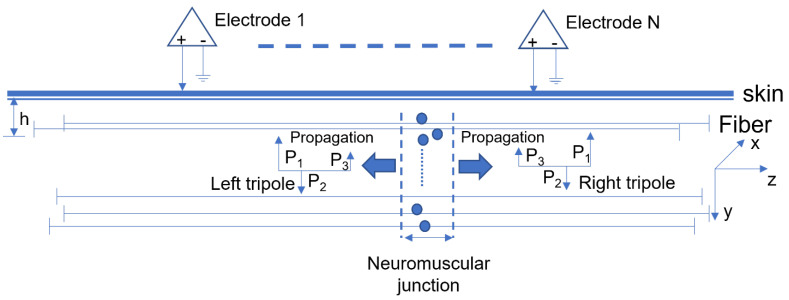
Model of fiber action potential generation and the detection system. All the muscle fibers are uniformly distributed in a cylinder at different depths h (y axis). A right and a left current tripole originate from the IZ and propagate at the direction of z axis to the fiber-tendon termination where they become extinguished.

The generation and extinction of MUAPs at the fiber endplate and tendon were considered. A tripole model described in [41] was used to simulate the generation of MUAP. Briefly, two action potentials generated by a fiber, modeled as two current tripoles, were originated at the IZ, propagated in opposite directions, and extincted at the fiber-tendon endings (Figure 1). The monopolar signal detected by the electrode is the summation of the contribution from each of the tripoles. An MUAP was simulated as the sum of its constituent fiber action potentials. For the muscle with one IZ, the neuromuscular junctions of all the MUs were approximately located at the mid-point of the muscle fiber. For the muscle with two IZs, the neuromuscular junctions of all the MUs were randomly distributed at two distinct regions along the muscle fiber direction. The neuromuscular junctions were uniformly distributed in a region of 1.5 mm width.

**Figure 2:**
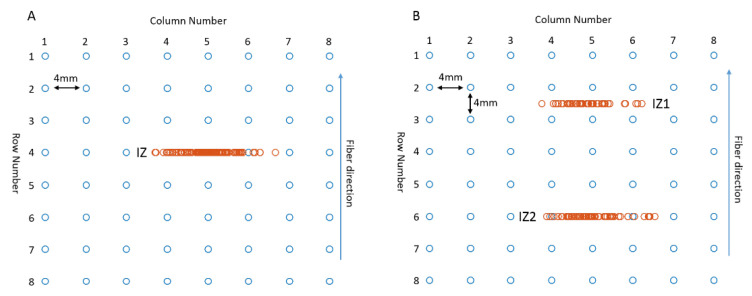
Simulated electrode matrix consisting of a grid with 8 columns (parallel to muscle fiber direction) and 8 rows. (**A**) The IZ was on row 4. (**B**) The two IZs were between row 2 and row 3, and at row 6, respectively.

As shown in Figure 2, the simulated surface EMG signals were recorded by a 64-channel surface electrode matrix (arranged in 8 by 8 channels, with an inter-electrode distance of 4 mm for both horizontal and vertical directions). The electrode matrix was placed with its columns aligned parallel to the muscle fiber direction. If the muscle was simulated to have one IZ, the IZ was simulated to locate at row 4. If the muscle was simulated to have two IZs, the first IZ (IZ1) was simulated to locate between row 2 and row 3, and the second IZ (IZ2) was simulated to locate at row 6. The distance between IZ1 and IZ2 was 14 mm.

Surface EMG signals from maximum voluntary contraction (MVC) were simulated. The simulated duration of contraction was 10 s. Surface EMG signals $x_{i}\left(t\right)$ at a specific channel i were generated as a sparse combination of MUAP trains from all 120 active MUs, as described in Equation (3):

(3) $$x_{i}\left(t\right)=\overset{N}{\underset{j=1}{\sum }}\overset{L-1}{\underset{\tau =0}{\sum }}a_{j}\left(\tau \right)s_{j}\left(t-\tau \right);\;$$

where $a_{j}$ is the MUAP waveform of the jth MU, L is the length of the waveform. $s_{j}\left(t\right)=\sum_{k}\delta \left(t-T_{j}\left(k\right)\right)$ indicates whether the jth MU discharges at a specific time t , where $T_{j}\left(k\right)$ is the kth discharge time of the jth MU and δ represents Dirac Delta function.

The M wave was simulated as a linear summation of MUAPs from all 120 active MUs. For both voluntary surface EMG and M waves, the signals were simulated at a sampling rate of 2 kHz per channel.

### 2.3. Motor Unit Synchronization

**Figure 3:**
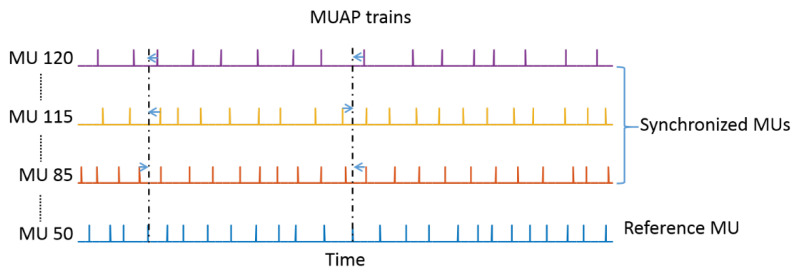
Schematic diagram of the MU synchronization. The MUAP trains discharged by 4 of the MUs are shown as examples. The timing of some impulses of the randomly selected synchronized MUs (MU120, MU115, and MU85) was adjusted to coincide with randomly selected impulses of the reference MU 50. This process was repeated 120 times so that each MU in the pool could be served as the reference MU.

To determine the effect of MU synchronization on IZ estimation, the timing of the independently generated MUAPs of some MUs was adjusted to impose a temporal association between some of the MUAPs discharged by other MUs. The synchronization process as described by Yao et al. [39] was used. Based on previous experimental reports, the level of synchrony was set at 15% in the simulation, meaning that that 15% of the impulses of each MU served as the reference to which 15% of the other active MUs had their impulses accordingly aligned. Briefly, four steps were involved in the process: (1) among all the active MUs, one MU was selected as a reference MU; (2) 15% of the MUAPs discharged by the reference MU were randomly selected; (3) among the rest of the active MUs, 15% of them were randomly selected as synchronized MUs; and (4) for each of the selected MUAPs of the reference MU, the timing of the nearest MUAPs of all the synchronized MUs were adjusted to be coincident with it. All the active MUs were selected as the reference MU in turn. When the adjustments were made for the reference MU, all the rest of the active MUs were candidates for the randomly selected synchronized MUs. A schematic diagram illustrating the synchronization process is shown in Figure 3.

### 2.4. IZ Location Estimation

The simulated monopolar signals of each column were post-processed to have single differential signals along muscle fibers. For M waves, the signals were visually inspected to estimate the IZ location as on the channel with the smallest signal amplitude or between the two adjacent channels whose signals show opposite polarity. For interference surface EMG where visually inspection might be uncertain, cross-correlation coefficients between adjacent channels were calculated to help estimate the IZ location.

### 2.5. Simulation Procedures

Muscles with one IZ and two IZs were simulated respectively, each with three conditions of MU synchronization. For the first condition, there was no synchronization with all MUs firing independently. For the second condition, a 15% level MU synchronization was imposed on the MU pool. The third condition was analog to the M-wave recording evoked by supramaximal electrical stimulation, where all MUs fired simultaneously. Because of stochastic components built into the model, 10 repetitions were simulated for each of the six situations, and the IZ estimation results were reported.

## 3. Results

### 3.1. Muscle with Single IZ

**Figure 4:**
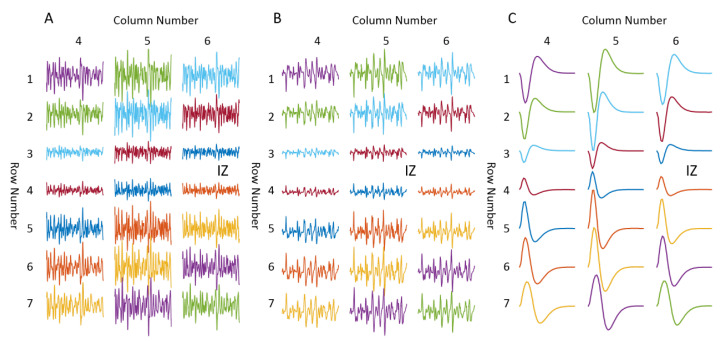
Example of IZ locations identified from simulated surface EMG of a single IZ muscle. (**A**) Interference surface EMG under no MU synchronization. (**B**) Interference surface EMG under 15% MU synchronization level. (**C**) M waves.

**Figure 5:**
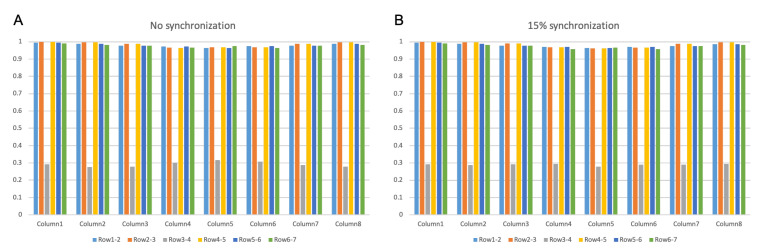
Correlation coefficients between the surface EMG signals of adjacent channels across all the 8 columns (for single IZ simulation). (**A**) For interference surface EMG under no MU synchronization. (**B**) For interference surface EMG under 15% MU synchronization level.

Figure 4 presents an example of the simulated differential surface EMG signals from column 4 to column 6, under different MU synchronization conditions, i.e., no synchronization, 15% level synchronization, and complete synchronization (M-wave recording), respectively. It can be visually observed, especially from the M-wave recording, that the signals at row 3 and 4 are of opposite polarity. Calculation of the correlation coefficients between adjacent channels of the interference surface EMG confirms that row 3 and row 4 had the minimum correlation coefficient across all the columns as shown in Figure 5, indicating that the IZ was approximately located between row 3 and row 4. In all the 10 repetitions of the simulation, the estimated IZs were consistently estimated between rows 3 and 4 across all the columns, regardless of the MU synchronization condition. In the case of single IZ in a muscle, the estimated IZ location from surface EMG agreed with the one used in the simulation.

### 3.2. Muscle with Double IZs

#### 3.2.1. IZ Estimation at No MU Synchronization

**Figure 6:**
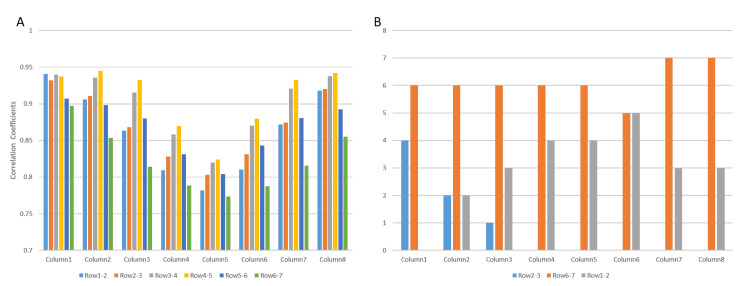
(**A**) Example of correlation coefficients between the signals from adjacent channels across all the 8 columns (for two-IZ simulation, no MU synchronization). (**B**) For each column, the counts of adjacent row pairs which had the minimum correlation coefficients in all the ten repetitions.

Figure 6A shows an example of the correlation coefficients between adjacent channels of the interference surface EMG for all the columns, derived from one repetition of the simulation in the case of no MU synchronization. It can be observed that row 6 and row 7 had the minimum correlation coefficients for all the columns, indicating that the IZ was approximately located between row 6 and row 7.

Figure 6B summarizes the results from 10 repetitions of simulation, where for each column the location of the minimum correlation coefficients across adjacent channels is presented, as well as the number of their occurrence out of the 10 repetitions. For example, for column 4, in 6 out of the 10 repetitions, the minimum correlation coefficient was found between row 6 and row 7, while for the other 4 repetitions, the minimum correlation coefficient was found between row 1 and row 2. As observed from the figure for all the columns, the minimum correlation coefficients were found either between row 6 and row 7, between row 1 and row 2, or between row 2 and row 3. Given the fact that in the model, the two IZs were located at row 2, and between row 6 and 7, the results indicate that at least one IZ can be approximately estimated.

#### 3.2.2. IZ Estimation with 15% MU Synchronization

**Figure 7:**
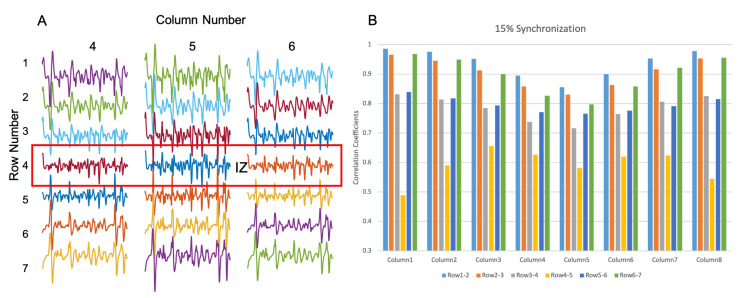
Example of IZ locations identified from simulated interference EMG with 15% synchronization level of a muscle with two IZs. (**A**) The simulated differential EMG signals. (**B**) Correlation coefficients between the signals from adjacent channels across all 8 columns (indicating the IZ was located between row 4 and row 5).

Figure 7A presents an example of simulated interference surface-EMG signals under 15% MU synchronization. The calculated correlation coefficients across adjacent channels are shown in Figure 7B, which indicates that row 4 and row 5 had the minimum correlation coefficient for all the columns. This suggests that the estimated IZ location was located between row 4 and row 5, which is different from either IZ location used in the simulation.

**Figure 8:**
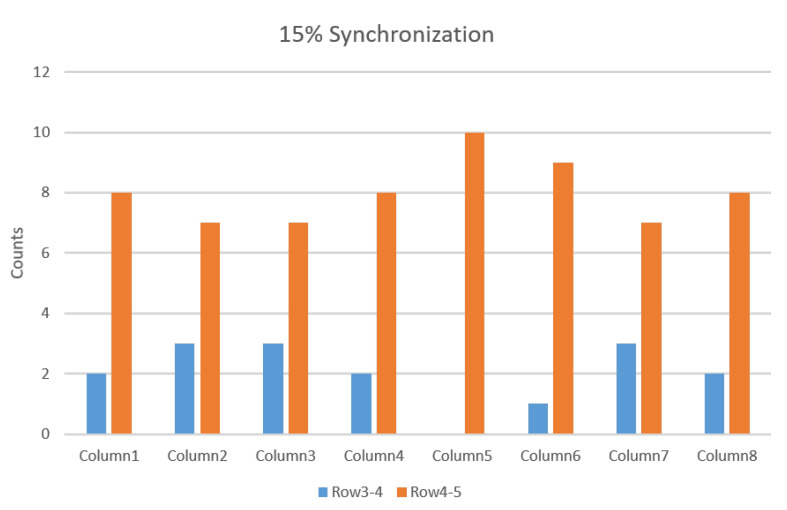
For each column, the counts of adjacent rows pair which had the minimum correlation coefficients in all the ten repetitions under 15% level synchronization (for two-IZ simulation). In most cases, the minimum correlation coefficients were found between row 4 and 5 or between row 3 and 4.

Similarly, Figure 8 summarizes the results from 10 repetitions of simulation with 15% MU synchronization, where for each column, the location of the minimum correlation coefficients across adjacent channels was presented, as well as the number of their occurrence out of the 10 repetitions. It was observed that the minimum correlation coefficients were found either between row 3 and row 4 or between row 4 and row 5. Given the fact that in the model, the two IZs were located at row 2 and between row 6 and 7, the IZ was not approximately estimated from interference surface EMG.

#### 3.2.3. IZ Estimation with M Waves

**Figure 9:**
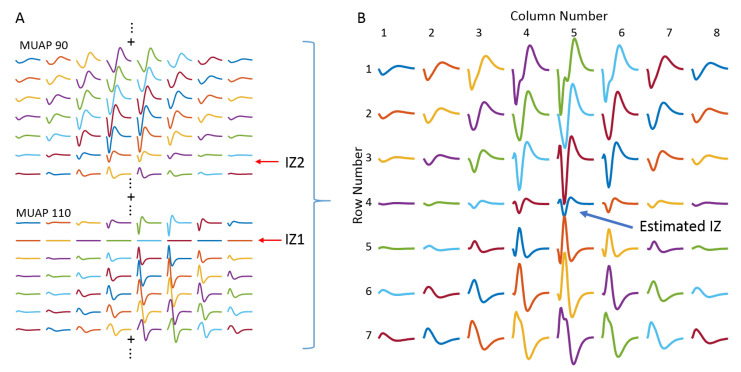
An example of IZ locations identified from simulated M wave of a two-IZ muscle. (**A**) The distribution of differential MUAPs. The simulated two IZs were located at row 2 and between row 6 and row 7, respectively. (**B**) The M wave generated from summation of all the MUAPs. The estimated IZ was around row 4.

Figure 9 shows an example of MUAP distributions across different channels and rows, and the resultant M-wave distribution from one repetition of the simulation. From the MUAP distributions, it demonstrates that the two simulated IZs were located at row 2 and between row 6 and row 7, respectively. However, from the M-wave distribution, it can be visually observed that row 4 had the least amplitude, and the signals from row 4 and row 5 were of opposite polarity, suggesting that the IZ was located at approximately row 4 or between row 4 and row 5. The same observations were found for all 10 repetitions of the simulation. Therefore, the estimated IZ location was not consistent with either one used in the model.

## 4. Discussion

The purpose of the present study was to evaluate the performance of surface-EMG-based IZ estimation with respect to different neurophysiological factors. The factors under investigation included muscles with single or double IZs, voluntary contractions with or without MU synchronization, and surface EMG signals generated from voluntary contraction or electrical stimulation. A computer model approach was used to simulate different situations by varying relevant model parameters which are otherwise difficult to manipulate experimentally. Computational models have been applied as a useful means for understanding electrical and mechanical outputs of a muscle in both healthy and pathological conditions [42,43,44,45,46]. In the present study, different situations regarding the number of IZs and the level of MU synchronization were simulated. When simulating MU synchronization during voluntary contraction, the amount of synchronization was comparable to that observed experimentally [39]. The precise MU firing strategy during voluntary contraction is still in debate, with both the so called “onion skin” and “reverse onion skin” firing strategies being reported in the literature [47,48,49]. In this study, only one firing strategy was simulated, since the difference in firing strategy is unlikely to have a significant impact on IZ estimation using the generated surface EMG signals.

The simulation results indicate that for a muscle with a single IZ, the IZ can be correctly estimated for all the tested conditions regardless of the level of MU synchronization (zero or 15% synchronization level) or the protocol used for surface EMG recording (voluntary EMG or M waves). Therefore, it can be concluded that MU synchronization has little effect on the performance of surface-EMG-based IZ estimation if there is only one IZ in the muscle. The simulation results also indicated that the same IZs were derived from the M waves and voluntary surface EMG signals, which is consistent with previous experimental findings [50,51]. For example, Guzmán-Venegas et al. reported that for the tibialis anterior muscle, no significant differences were found between the IZ locations estimated from the two protocols of electrical stimulation and voluntary contraction [50]. Huang et al. also reported a substantial concordance between the locations of the IZs estimated by M waves and interference surface EMG under relatively low contraction levels (5–40% MVC), while the identified IZ tended to be more proximal during strong voluntary contractions (60–100% MVC) [51]. This could be likely due to muscle shortening induced by strong contractions, which was not considered in our simulation.

The most interesting finding of the study is from the simulation of the muscles with two IZs, where MU synchronization appears to have a dramatic influence on the performance of IZ estimation. Although not a common observation for a superficial muscle having multiple IZs it has been reported in some individuals among the tested subjects [1,11,31,32]. For muscles with two IZs, the simulation results indicate that it is likely to estimate at least one of the two IZs when there is no MU synchronization. However, with the presence of MU synchronization, none of the two IZs can be correctly estimated. Instead, a false IZ (not consistent with the one used in the model) was estimated based on interference surface EMG signal processing. A similar IZ estimation outcome was achieved from M-wave recordings when all MUs were synchronized by supramaximal electrical stimulation. This suggests that MU synchronization plays an essential role in affecting the IZ estimation when a muscle has two IZs. A previous simulation study has shown that under MU synchronization, the MUAPs from different MUs tended to coincidentally overlap, which increased the magnitude of the average rectified EMG (and the variability in the simulated force) [39]. It is noted that this finding is from a single IZ muscle. However, when the synchronized MUs have different IZ locations, the synchronization increases the degree of negative and positive phase cancellation for the bipolar electrode configuration between the two IZ regions. This will affect the generated surface EMG signals, further leading to incorrect IZ identification. The effect of MU synchronization imposed to surface EMG can be more straightforwardly demonstrated by the M waves, as shown in Figure 8. It can be observed from the figure that the M waves at row 2 are mainly from MUAPs originated from IZ2, making IZ1 difficult to be identified. For the bipolar electrode configuration between IZ1 and IZ2 (such as row 4 and row 5), the positive and negative phases of MUAPs originating from the two different IZs would overlap, leading to significantly reduced EMG amplitude and phase reversal (because of signal cancellation). As a result, a false IZ would be identified based on surface EMG signals.

The findings of the study indicate that caution is required for IZ estimation when a muscle has multiple IZs, particularly when surface EMG signals are recorded from a situation where MU synchronization is often reported, such as in various neuromuscular pathologies [52,53], during exercise training [54,55], during specific muscle contraction paradigms or tasks [56,57], during muscle fatigue [58,59], etc. Of particular note, determining the IZ location can help optimize the botulinum neurotoxins injection in clinical practice of spasticity treatment [6]. For patients who are unable to perform voluntary muscle contractions because of paralysis or impaired motor control, recording of M waves can be used as an alternative approach for IZ estimation. However, there is a potential disadvantage of false IZ estimation for muscles with multiple IZs (such as biceps brachii and brachioradialis muscles [11]).

Considering the practical circumstances of muscle IZ estimation, the relevant information (such as the number of IZs, the level of MU synchronization) is not known a priori. Therefore, it remains a dilemma in assessing the performance of IZ estimation using voluntary surface EMG or M waves. To solve this difficulty, a useful strategy is to obtain individual MU contributions to the surface EMG, either by surface EMG decomposition or incremental stimulation. With advances in surface EMG decomposition, it is feasible to obtain individual MUAP trains from high-density surface EMG [60,61,62]. The spatial distribution of MUAP templates across the electrode array can provide useful information for identifying the MU IZ. If a relatively large number of MUs can be extracted, their IZ distribution will provide a general picture (such as the number and locations) of the muscle IZs. Meanwhile, the MU firing behavior obtained from EMG decomposition can be used to assess the level of MU synchronization.

As a model-based computer simulation study, the limitations should always be acknowledged. Various assumptions were used in the model, while some neurophysiological factors were not thoroughly considered in the simulation, such as different MU locations, depth, territory, or muscle fiber size. Instead, simplifications were applied; for example, all the MUs were assigned the same muscle fiber diameter, and muscle fibers of each MU were widely scattered throughout the whole muscle. Previous studies have reported that MU synchronization increased significantly with the mean recruitment threshold of MUs [63], while our simulation did not account for this relation. In simulation of M waves, the time delay of different MUAPs arriving at the recording electrode was not considered. Simplification was also applied to IZ simulation, where two narrow bands perpendicular to muscle fibers were designated as IZ. However, muscle IZ distribution may be of more complexed patterns [64]. To address these issues, a more delicate or realistic model is required in a future study. In addition, only conventional IZ estimation methods based on visual inspection, amplitude measurement and cross-correlation were used in this study, while more advanced methods for IZ estimation were not tested [21,22,23,24,25,26,27,28,29].

In summary, with the current model, we investigated different neurophysiological factors on muscle IZ estimation using surface EMG signals. The results indicated that MU synchronization had little effect on IZ estimation if there was only one IZ in the muscle, which can be approximately estimated using either voluntary surface EMG or M-wave recordings. However, when a muscle had two IZs, the estimated IZ was not consistent with any of the IZs used in the model. This was observed for both M waves and voluntary surface EMG signals with MU synchronization, suggesting that false IZ estimation might occur in the case of two IZs and MU synchronization. These findings provide important clues for understanding and avoiding pitfalls during IZ estimation using surface EMG.

## Data Availability

The simulated data of this study are available on request from the corresponding author (PZ).
